# Boundary coding in the rat subiculum

**DOI:** 10.1098/rstb.2012.0514

**Published:** 2014-02-05

**Authors:** Sarah Stewart, Ali Jeewajee, Thomas J. Wills, Neil Burgess, Colin Lever

**Affiliations:** 1 Institute of Psychological Sciences, University of Leeds, Leeds LS2 9JT, UK; 2 Department of Cell and Developmental Biology, UCL, London WC1E 6BT, UK; 3 Institute of Cognitive Neuroscience, UCL, London WC1N 3AR, UK; 4 Institute of Neurology, UCL, London WC1N 3BG, UK; 5 Department of Psychology, University of Durham, Durham DH1 3LE, UK

**Keywords:** boundary vector cell, border cell, boundary-off cell, subicular, wall, edge

## Abstract

The spatial mapping function of the hippocampal formation is likely derived from two sets of information: one based on the external environment and the other based on self-motion. Here, we further characterize ‘boundary vector cells’ (BVCs) in the rat subiculum, which code space relative to one type of cue in the external environment: boundaries. We find that the majority of cells with fields near the perimeter of a walled environment exhibit an additional firing field when an upright barrier is inserted into the walled environment in a manner predicted by the BVC model. We use this property of field repetition as a heuristic measure to define BVCs, and characterize their spatial and temporal properties. In further tests, we find that subicular BVCs typically treat drop edges similarly to walls, including exhibiting field repetition when additional drop-type boundaries are added to the testing environment. In other words, BVCs treat both kinds of edge as environmental boundaries, despite their dissimilar sensory properties. Finally, we also report the existence of ‘boundary-off cells’, a new class of boundary-coding cells. These cells fire everywhere except where a given BVC might fire.

## 1. Introduction

The spatial mapping function of the hippocampal formation [1,2] is likely derived from two sets of information: one based on the external environment and the other based on self-motion. In this report, we focus on how a certain type of spatial cell responds to changes in environmental boundaries.

In order to explain spatial features of place cell firing, such as place fields stretching when an environment is expanded [3], and the shapes of place fields across environments which differ only in shape [4], ‘boundary vector cells’ (BVCs) were predicted as inputs to place cells [3,5–8]. A BVC would fire whenever an environmental boundary intersected a receptive field located at a specific distance from the rat in a specific allocentric direction, with breadth of tuning to distance that increases with the preferred distance. The firing of model BVCs depends solely on the rat's location relative to environmental boundaries and is independent of the rat's heading direction. The firing of a place cell can be modelled as a thresholded sum of the firing of the BVCs synapsing onto it, and the BVC model captures several features of place fields in different environmental configurations [5].

The BVC model followed early reports of the importance of environmental boundaries for the firing of place cells [9,10]. An aspect of the BVC model is that it separates the functional significance of different types of cues. Distal cues (at or beyond the edge of the environment) provide an allocentric directional reference frame, presumably mediated by head direction cells [11], within which the directional preferences of BVC tuning curves are encoded. Once the directional reference frame is established, a given environmental location (and the firing of a place cell at that location) is encoded in terms of the conjunction of distances to boundaries along these preferred directions. The model thus also provides a mechanistic explanation for how the firing patterns of place cells are deformed by manipulations of directional cues [12], as described in [13,14]. In addition, the BVC model implies that the locations of place cell firing are determined by the proximal boundaries of the animal, thus explaining why place cell firing fields tend to maintain their location relative to the behavioural testing box when it is moved within the testing room [3,4,15].

The discovery of BVCs in the subiculum was first reported in [16] and then described more comprehensively in [17]. However, the subiculum is typically regarded as an output region of the hippocampal formation [18–20], constraining views on how boundary cells might input to place cells in the hippocampus proper. (We suggest a more complex view of the subiculum by noting, e.g. physiological evidence that subicular output can enter the hippocampus proper indirectly [21] and that CA1 place cells are not obviously influential on subicular firing in many situations; full discussion of this is beyond the scope of this brief report.) Additional support for the BVC model was thus provided by the discovery of border/boundary cells in the medial entorhinal cortex [22,23], which receives a prominent input from the subiculum, and which projects monosynaptically to hippocampal place cells. It has also been shown that the presubiculum and parasubiculum, which receive input from the subiculum and project to the entorhinal cortex, contain border/boundary cells, as well as grid cells [24]. The presence of both boundary cells and grid cells in regions around the hippocampus proper strongly suggests the importance of both external environmental cues and self-motion cues in the generation of accurate and stable spatial coding in the hippocampus.

In this report, we describe two types of environmental manipulation: the introduction of an internal upright barrier and the introduction of drop-type boundaries. In both types of manipulation, we examine the phenomenon of field repetition, where the boundary cell exhibits an extra field when an additional, appropriately oriented, boundary is created within the testing environment. This manipulation provides a strong test of the BVC model. It builds on the prediction, and subsequent demonstration, of second fields in some place cells in response to barrier insertion [5,7], driven by presumed BVC inputs. As the environment becomes familiar, plasticity in the model's BVC to place cell connections causes a ‘tidying’ of place cell firing, such that regions of lower firing rate are lost while regions of higher firing rate strengthen [14], consistent with experimental data from CA1 [4,16,25,26]. This plasticity also provides an explanation for the appearance of place fields that respond in a single location relative to a barrier, after experience of the movement of the barrier relative to the environment [26], as described in [14]. The BVC model predicts that these cells initially had firing fields both at the barrier and at the edge of the environment.

The theoretical descriptions of BVCs to date [5,6,14,17,27] have largely been tailored to walled environments (although more general mechanisms of detecting distances to boundaries have been considered, such as the angle to the edge of the floor [8] or optic flow [27]). However, many environmental boundaries in natural and man-made environments are drops (e.g. cliff-edges on land and rock, table tops). Thus, it is important to understand whether drops can elicit field repetition in BVCs, and thus whether BVCs treat drops similarly to walls. As walls (i.e. continuous vertical surfaces) and drop edges have very different sensory representations, similar coding of walls and drops would further underline the idea that BVCs are specialized to code for environmental boundaries *per se*.

## 2. Material and methods

### (a) Animals

Five naive adult male Lister Hooded rats weighing 330–400 g at the time of surgery were maintained on a 12 L : 12 D schedule, lights off at 13.00. After surgery, they were housed individually and kept at 85% free-feeding bodyweight. All procedures complied with the UK Animals (Scientific Procedures) Act 1986.

### (b) Surgery and implants

Under deep anaesthesia, rats were chronically implanted with two microdrives above dorsal subiculum or other hippocampal regions, one per hemisphere. Each microdrive allowed a bundle of four movable tetrodes to be vertically lowered through the brain after surgery. Tetrodes were constructed from four twisted 25 µm HM-L-coated platinum/iridium (90%/10%) wire (California Fine Wire, Grover Beach, CA, USA). Skull coordinates (relative to bregma) for subiculum implants targeted anterior locations (5.4 AP, 1.6–2.0 ML) or posterior locations (6.2–6.4 AP, 3.2–3.4 ML).

### (c) Data acquisition

From about one week after surgery, tetrodes were gradually lowered over days and weeks towards the subiculum. Electrophysiological screening took place while the rat was on a holding platform within the testing laboratory. Electrophysiological recording was carried out as described in [17,28]. Briefly, electrical signals were acquired at 250 Hz (local field potentials) and 50 kHz (single-cells) via a 32-channel or 64-channel system (Axona, St Albans, UK). They were bandpass filtered at 300 Hz–7 kHz for single-cells, after having been amplified approximately 10 000–20 000 times. Position data were sampled at 50 Hz using light-emitting diodes. Speeds above 2 m s^−1^ were discarded, because they were likely to have resulted from head movement or light reflection.

### (d) Environments and trials

Rats were placed on a holding platform between trials. Probe trials were run generally in between two baseline trials. Baseline trials were run in either the walled circle or the walled square environment. Both environments used the unwalled circle (155 cm diameter black platform, elevated 30 cm from the laboratory floor) as the base. The walled circle environment (150 cm diameter) had white 50 cm high walls. The walled square environment (100 × 100 cm) had black 50 cm high walls. The inserted barrier was 50 cm high, 50 cm wide and 3 cm thick. This inserted barrier was painted black (the same as the walls of the walled square).

The ‘together–apart’ manipulation consisted of three 50 × 50 cm black square open platforms (elevated 50 cm from the laboratory floor). In the ‘together’ trial, these were tightly juxtaposed to create a 150 × 50 cm rectangular open platform. In the ‘apart’ trial, the three-square platforms were separated by 10 cm to create two traversable gaps between the platforms.

### (e) Boundary vector cell sampling procedure

**Figure 1.**
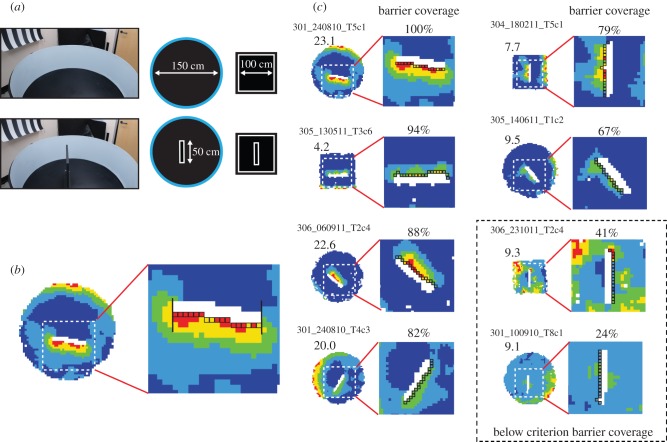
The procedure used to classify boundary cells as BVCs. (*a*) Photo and diagram of the barrier insertion manipulation. An upright barrier (50 cm long, 50 cm high, 3 cm thick, painted black matt) was inserted into either the walled circular environment (150 cm diameter), which had light matt walls, or the walled square environment (100 × 100 cm), which had black matt walls. (*b*) Firing rate map for a cell in a barrier trial whose second field extended along the entire length of predicted side of the barrier. White dashed box within rate map (left) and red lines indicate the area around the inserted barrier where the second field occurs, depicted in the zoomed-in firing rate map (right). The criterion for a cell to be classified as a BVC was that firing was required to be at least 40% of the locational peak firing rate along 50% or more of the bins directly abutting the predicted side of the barrier. The bins are indicated on the close-up as black-sided squares. (*c*) Representative examples of cells which did (*n* = 6 shown), and did not (*n* = 2 shown inside dashed box), meet the BVC field repetition criterion described in (*b*). For each cell, as in (*b*), we show whole-environment rate map (left), and close-up of the region containing the second field (right). Locational peak rate in hertz is shown top left of whole-environment rate map in this and subsequent figures. The proportion of barrier coverage (i.e. percentage coverage of second field along predicted side of the barrier) is shown above the close-up rate map. For instance, ‘67%’ indicates that firing rate was at least 40% of the peak firing rate in two-thirds of the bins along the length of the inserted barrier. 42/46 BVCs were classified using this criterion.

The typical procedure for identifying BVCs was first to identify a cell firing at or near the perimeter of the walled circle or walled square (baseline trial). Then, an experimental barrier was placed in the central region of the walled environment, oriented such that it was perpendicular to the presumed preferred direction of the boundary cell (barrier trial). The environments are shown in figure 1*a*. Appropriate field doubling was then used as a heuristic measure to classify the cell as a BVC. For instance, if a cell fired along the south wall of the walled square, a barrier would be placed in a west–east orientation: it would then be predicted that the cell would exhibit an additional firing field along the north side of the barrier, because there was now another region which satisfied the condition that there was a proximal boundary to the south of the rat. For each cell, the number of bins was counted along the length of the predicted side of the barrier (figure 1*b*). The threshold for a sufficiently robust second field was as follows: when the number of bins with firing rate at least 40% of peak rate occupied 50% or more of the length of the barrier. Figure 1*c* shows six examples of above threshold second fields, with the percentage of barrier coverage indicated above the rate map of the peri-barrier region. The dashed box shows two examples of second fields which did not meet our threshold criterion.

### (f) Analyses of spatial firing

Spatial analyses of boundary and head direction cells were conducted on locational firing rate maps and polar plots constructed as follows, except where otherwise stated (see §2*g*). Locational firing rate maps were constructed from locational bins each approximately 3 × 3 cm in size, smoothed using a 5 × 5 bin boxcar filter. Spike count divided by dwell time gave firing rate per bin. Firing rate maps are autoscaled false colour maps, each colour representing a 20% band of peak firing rate, from dark blue (0–20%) to red (80–100%). Directional firing polar plots were constructed from approximately 6° bins, each bin being smoothed by the two bins around it in both directions. Locational (directional) peak rates are the highest firing rate after smoothing shown in any locational (directional) bin and are always shown above left of the firing rate map (polar plot). Locational (directional) selectivity [17,29] was locational (directional) peak rate divided by global mean firing rate. Spatial information (locational, directional) was calculated in bits per second according to the formula in [30].

### (g) Correcting for inhomogeneous sampling

**Figure 2.**
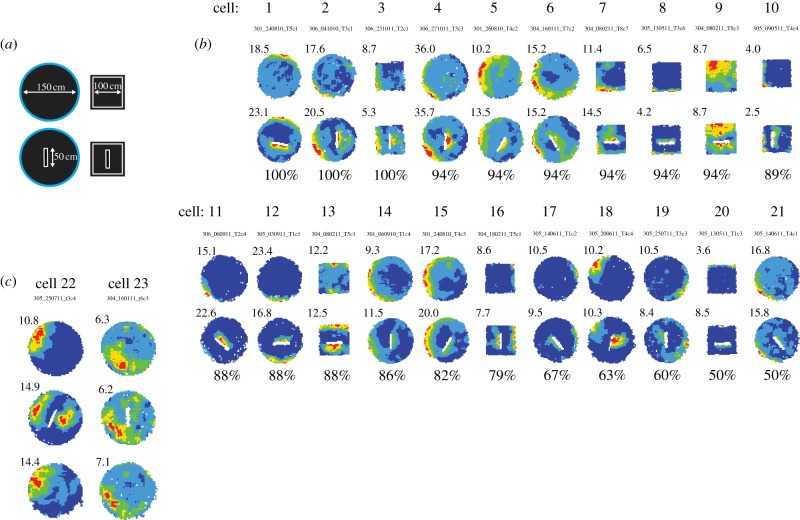
Examples of cells showing barrier-elicited field repetition predicted by the BVC model. (*a*) Diagram of environments used. (*b*) Firing rate maps of 21 BVCs with fields close to the walls and inserted barrier (i.e. short-range distance tuning). Locational peak rate in hertz is shown top left of firing rate map. Barrier coverage (i.e. percentage coverage of second field along predicted side of the barrier, see figure 1 and §2*e*) is shown below the firing rate map of the inserted-barrier trial. (*c*) Firing rate maps of two BVCs with fields further away from the walls and barrier (i.e. longer range distance tuning).

**Figure 3.**
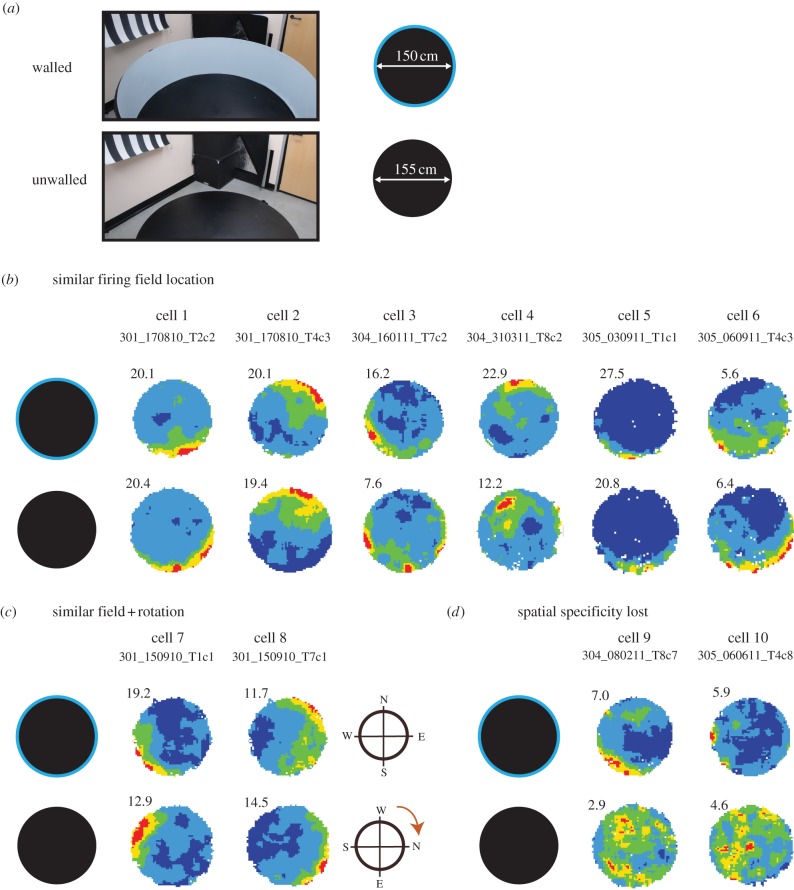
Most BVCs treat drops like walled boundaries. (*a*) Photo and diagram of the wall removal manipulation. The rat was placed on the holding platform between trials. The rats were tested in the walled circular environment (150 cm diameter); then the walls were removed and they were then tested on the unwalled circular platform (155 cm diameter). The rat was placed on the holding platform between trials. Fifteen BVCs were recorded in this manipulation. (*b*) Six examples of BVCs which showed similar spatial firing in both the walled circle and the unwalled circle. (*c*) Two examples of BVCs where fields rotated upon wall removal (here approximately 90° clockwise). (*d*) Two examples of BVCs where spatial specificity was lost upon wall removal.

**Figure 4.**
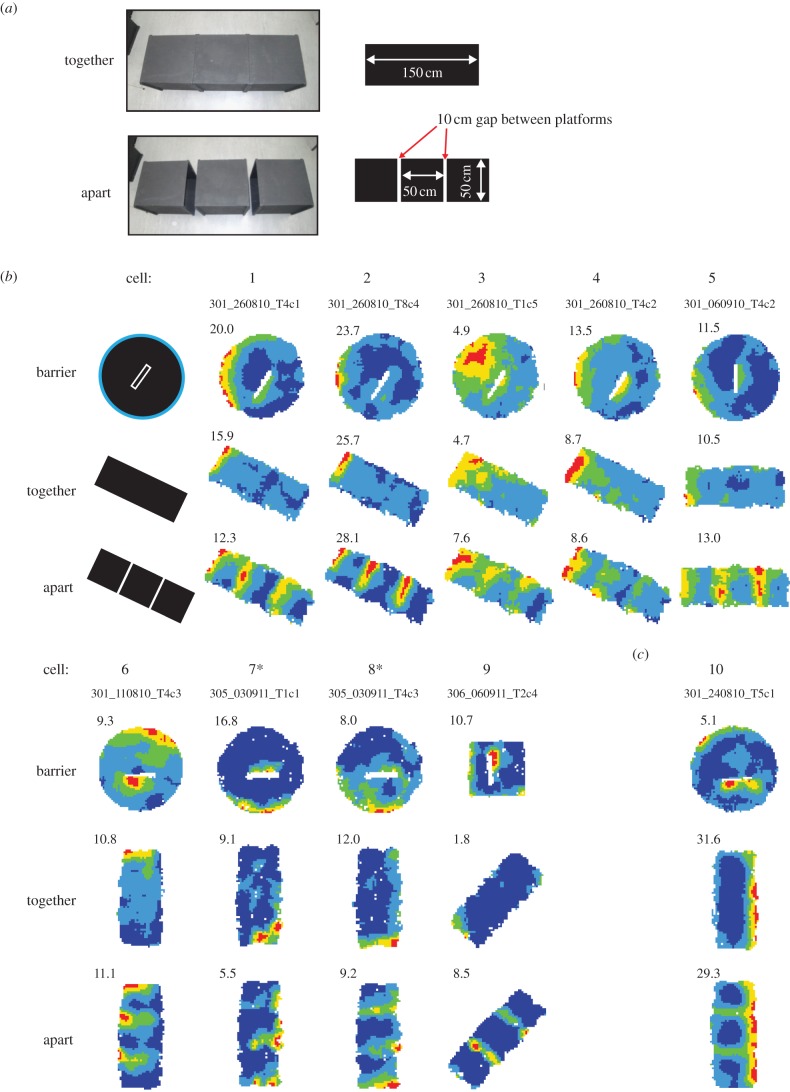
Field repetition in BVCs to drop-type environmental boundaries. (*a*) This manipulation included two trials, the ‘together’ trial, in a rectangular open platform made up of three square platforms, followed by the ‘apart’ trial where the platforms were separated by two 10 cm gaps. The environment was orientated so that one of the short edges was in the BVC's preferred direction. (*b*) Responses of nine BVCs which afforded a test of drop-elicited field repetition, i.e. exhibited a single restricted field in the ‘together’ trial such that the orientation of the long axis of the three square array was perpendicular to the long axis of the single field. All nine cells exhibit predicted field repetition in the ‘apart’ configuration. Barrier trials are shown for reference, top rows. (*c*) Example of a BVC where the long axis of the three box array was not oriented in line with the angular tuning preference of the cell but which showed additional fields at the traversable drops in the ‘apart’ configuration.

**Figure 5.**
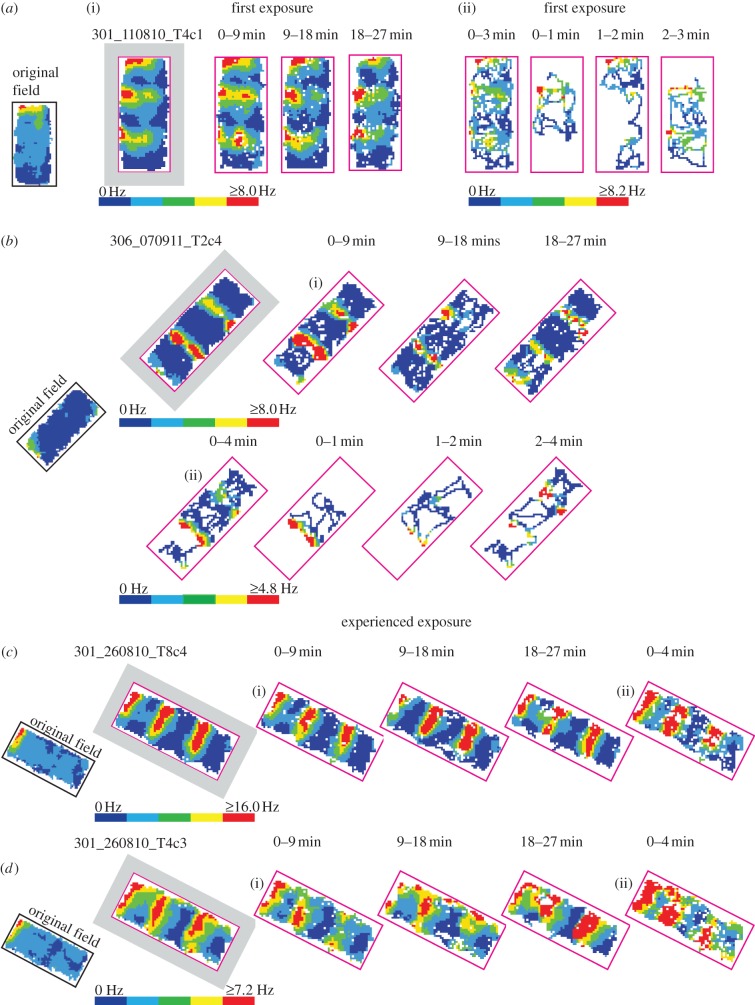
No sign of experience-dependent change in field-repetition response to drop-type environmental boundaries. No sign that experience shapes field repetition: (*a*,*b*) additional firing fields elicited by newly created environmental boundaries are observed immediately upon *the very first exposure* to the ‘apart’ configuration; (*c*,*d*) neither original nor duplicate fields weaken/disappear after environmental experience (eighth exposure, two weeks after first exposure, same rat as in *b*). Field repetition is clearly visible in the first, middle and last 9-min segments of every trial (a(*i*), *b*(i), *c*(i) and *d*(i)) and in the first 3/4 min of every trial (*a*(ii), *b*(ii), *c*(ii) and *d*(ii)). Rats were always initially placed in the middle one of three square platforms. Thus, firing fields observed in the very first minute during the very first exposure (*a*(ii), *b*(ii): ‘0–1 min’) were new ‘duplicate’ fields in response to the newly created boundary. In all parts of the figure: leftmost ‘original field’ thumbnail shows rate map in ‘together’ configuration prior to ‘apart’ configuration; grey-surround indicates whole-trial rate map, with other rate maps showing time-limited trial segments; pink rectangular-surround shows spatial limits common to all trial segments.

To directly compare locational versus directional signalling in BVCs, we applied the procedure in [31] to correct for spurious dependencies created by inhomogeneous sampling of orientation and location (see [29] and [17] for related spatial cell analyses). As noted in [31], inhomogeneity of sampling is unavoidable in freely moving animals and is often particularly acute at the boundaries of an environment, where locations can only be approached in particular directions. Corrected locational firing was calculated from unsmoothed firing rate maps. Corrected directional firing was calculated using unsmoothed polar plots. Note that locational bin sizes are appreciably larger than those used in the firing rate maps shown in figures 1–5, and in the electronic supplementary material, figure S1. As absolute information values are typically highly dependent on bin number, it is important to match the number of bins for locational and directional activity. This was achieved by selecting a (large) locational bin size such that the number of visited locational bins in the testing environment (60.3 ± 1.14 locational bins) was very close to the number of directional bins (exactly 60). The resulting bin sizes used in corrected analyses were as follows. Directional bins: 6°; locational bins: 18.5 × 18.5 cm in the 150 cm-diameter circle (*n* = 40 cells) and 14 × 14 cm in the 100 × 100 cm square environment (*n* = 6 cells).

### (h) Monte Carlo simulation of field peaks

To determine whether the distance of field peaks from the centre of the environment in 15 cells was greater than expected by chance, a Monte Carlo method was used. A null population of mean random distances to the environment centre was generated on the basis of the assumption that peaks were randomly distributed within the two-dimensional space defined by the recorded arena. To do this, 15 random positions were generated, and the mean distance to the environment centre calculated. This process was repeated 1 000 000 times, after which the values for the mean, median and 95th percentile of the null population had converged to two decimal places. The radius of the unwalled circle was behaviourally defined as 81 cm, that is, 3.5 cm longer than the radius to the perimeter edge (77.5 cm).

### (i) Analyses of temporal firing characteristics

Global mean rate was the number of spikes divided by the trial length (in seconds). Theta modulation was calculated as described in [17]. Briefly, the power spectrum of each cell's spike-train 500 ms autocorrelogram, based on spikes obtained during runs of at least 0.5 s, when the rat's speed exceeded 5 cm s^−1^, was used to assess the extent to which each individual cell's spiking was modulated by theta. The theta-modulation score gives the ratio of the average power in a narrow band (2 Hz) centred on the peak in the theta range (6–12 Hz) to the total average power in the whole spectrum (0–125 Hz). (This theta peak value was not predetermined but varied across cells and rats.)

## 3. Results

In this report, we characterize the spatial correlates and other firing properties of 46 BVCs. To provide a comparison sample for boundary cells, we also report on 30 head direction cells [11]. Importantly, these were recorded in exactly the same environments (from the subiculum and neighbouring regions).

### (a) Walled boundaries: barrier-elicited field repetition

The typical procedure (see further description in §2*e* and figure 1) for identifying BVCs was first to identify a cell firing at/near the perimeter of the walled circle or walled square. Then, we placed an appropriately oriented experimental barrier in the central region of the walled environment (barrier trial). The environments are shown in figure 2*a*. Appropriate field doubling was then used as a heuristic measure to classify the cell as a BVC. In most cases, firing field peaks were close to the walls. As defined by the criterion for barrier-elicited fields, an additional field was elicited by the barrier in 74% (42 out of 57) of the cells. Figure 2*b* shows baseline and barrier trials for half of this sample (*n* = 21) in descending order (100–50%) of the spatial extent of firing along the barrier. Four additional cells were also classified as BVCs: two cells with perimeter- and barrier-elicited fields located away from the walls (figure 2*c*); and two cells whose barrier-related firing did not meet the criterion for a barrier-elicited additional field, but did show drop-elicited field repetition (see figure 4*b*). Thus in total, 46 cells were classified as BVCs. Electronic supplementary material, figure S1 shows locational rate maps, directional polar plots, waveforms and 500 ms temporal autocorrelograms for all 46 cells.

In summary, a high proportion of subicular cells which fire near walled perimeters fire in a manner predicted by the BVC model when internal barriers are placed within the environment.

### (b) Unwalled boundaries: drop-related firing

We employed two tests of drop-type boundaries. In the first test, after a baseline trial in the standard walled circle, the walls were removed, leaving the elevated circular floor as an additional testing environment (‘unwalled circle’). In the second test, referred to as the ‘together–apart’ manipulation, three elevated squares were placed in a linear array, either tightly juxtaposed (‘together’) or with 10 cm gaps between them (‘apart’). A key aim here was to test whether the phenomenon of field repetition, seen in barrier-elicited second fields in the walled environments, would extend to drop-type boundaries. These two tests were conducted on overlapping subsets of BVCs.

In the first test, we compared firing in the walled versus unwalled circle (figure 3*a*). The average distance of BVC locational field peaks from the centre of the *walled* circle was 70.74 ± 1.26 cm. In other words, BVC locational field peaks were located very near to the boundary of this walled circle (on average approximately 4 cm from the boundary). If these cells were specialized to code for environmental boundaries, one would predict that the locational field peaks of these cells would also be located near the drop-type boundary in the *unwalled* circle. The results confirmed this prediction. Generally, BVCs' firing field peaks continued to occur near the edges of this drop-type probe environment. The average distance of the BVC locational field peaks from the centre of the *unwalled* circle was similar to that in the walled circle (66.59 ± 4.76 versus 70.74 ± 1.26 cm; paired *t*_14_ = 0.84, *p* = 0.42). Defining the outer portion of the environment as the region between the perimeter and the circle obtained at 75% of the radial distance, 80% (12 out of 15) of the BVCs’ firing field peaks occurred in the outer portion. Examining the distribution of the BVC field peaks in the unwalled circle (we take into account its slightly larger size—see §2*h*), we note that the mean distance of the peaks from the centre is very unlikely by chance, assuming a random distribution of field peaks over the area of this circle. Monte Carlo simulation showed that the mean distance from the centre of the 15 BVC field peaks in the unwalled circle (66.59 cm) lay on the 99.7th percentile of the mean distance from the centre of 15 randomly distributed peaks (i.e. *p* < 0.005). In summary, as a whole, BVCs continued to show boundary responsive coding in the unwalled circle.

The most common response of BVCs in the unwalled circle was to fire in a similar region as that in the walled circle (e.g. figure 3*b*, cells 1–6). For a few cells (figure 3*c*), the field appeared to have rotated. For instance, cell 7 fired along the southwestern portion of the walled circle, but fired along the northwestern portion in the unwalled circle, suggesting a clockwise rotation of the head direction (HD) system by approximately 90° (figure 3*c*, see also cell 8, which was simultaneously recorded with it). (Although our aim was to ‘clamp’ the directional system, this was likely not always successful.) Four of the 15 BVCs lost spatial specificity in the unwalled circle (figure 3*d*), suggesting that these cells required continuous vertical surfaces (i.e. walls) for precise boundary coding.

Figure 4 shows the results of the second test, which we call the ‘together–apart’ manipulation. Figure 4*a* illustrates the three square environments used in this manipulation: first, the tightly juxtaposed configuration (‘together’, top row), and then in the configuration with 10 cm gaps between the squares (‘apart’, bottom row), which the rats were able to cross without any assistance. In all, nine cells afforded a test of drop-elicited field repetition; that is, in the ‘together’ configuration, these cells exhibited a single restricted field and the orientation of the long axis of the three square array was perpendicular to the long axis of the single field. As figure 4*b* shows, importantly, all of these nine cells exhibited the predicted field repetition in the ‘apart’ condition, elicited by the addition of two drop-type boundaries. (Barrier trials for these cells are shown for reference; figure 4*b*, top rows.) Figure 4*c* shows an example of a BVC whose angular tuning preference presumably rotated between the baseline and ‘together–apart’ configurations such that the long axis of the three-box array was not perpendicular to the angular tuning preference of the cell. Interestingly, however, the cell showed additional fields at the traversable drops in the ‘apart’ configuration.

### (c) No sign of experience-dependent change in field repetition to boundaries

Some insight into the mechanisms of boundary coding might be afforded by understanding the role of experience in shaping the response to boundaries. Actually, we have seen no sign that experience shapes field repetition. Figure 2 shows barrier-elicited field repetition at various stages of experience of the inserted barrier condition. In this section, we present evidence relating to the dynamics of field repetition to drop-type boundaries. Do perhaps the cells require that the rat at least moderately experiences each of the three drop-type boundaries in a testing environment before they are able to fire similarly across all of them? Actually, we see no evidence in favour of this view. Figure 5 shows responses of four of the nine BVCs in figure 4 in closer temporal detail. Figure 5*a*,*b* shows two cells from two different rats firing in predictable ‘duplicate’ regions additional to the original field upon the very first exposures to the ‘apart’ configuration. These new duplicate fields are seen at the earliest sampling opportunity in the very first minute during these very first exposures (figure 5*a*(ii),*b*(ii)). In a complementary fashion, figure 5*c* shows two simultaneously recorded cells in the given rat's eighth exposure to the ‘apart’ configuration, two weeks after the first exposure. It seems clear neither the original nor duplicate fields weaken/disappear after experience (as often occurs in place cells which show field repetition, see §4 for more details). Of course, we cannot rule out subtle differences, nor have we tested the effects of heavy training in these environments.

In summary, initial environment-specific experience does *not* seem to be required to establish field repetition and environment-specific experience does not diminish field repetition.

### (d) ‘Boundary-off’ cells

Using barrier-elicited field repetition as a heuristic to define BVCs, we note that some BVCs have presumptive interneuron-like waveforms (see the electronic supplementary material, figure S1). In other words, in those regions of the environment where these presumptive interneuron BVCs fire, the cells would act to *inhibit* the firing of their efferent target neurons. Such a hypothetical scenario may help to explain the phenomenon of ‘boundary-off’ cells described in this section.

**Figure 6.**
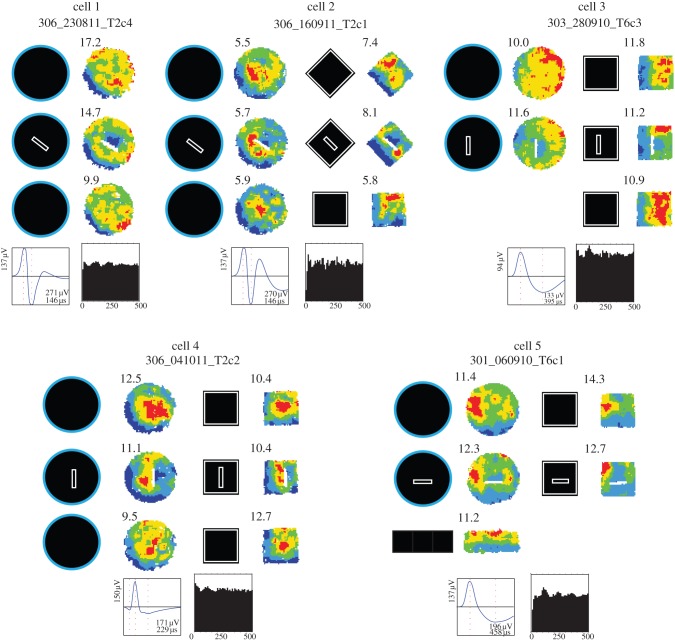
Boundary-off cells. Five examples of boundary-off cells recorded in baseline and barrier trials. Below the rate maps is the spike waveform, bottom-right text stating peak amplitude (the highest positive-to-negative or negative-to-positive amplitude, µV) and negative peak-to-trough interval (µs) and the temporal autocorrelations (0–500 ms). Waveform and theta-modulation data come from the first large walled circle trial of the day.

Boundary-off cells fire more or less everywhere *except* where a given BVC might be expected to fire. Figure 6 shows the five clearest examples of boundary-off cells.

In the baseline condition (figure 6, top row of firing rate maps), there is a clear zone of markedly lower firing rate. For cells 1, 2 and 4, for instance, the low-firing zone is along the southwest perimeter of the walled circle. In general, the spatial firing pattern resembles the inverse of that of a short-range BVC. Thus, cells 1, 2 and 4 fire more or less everywhere except when a proximal boundary occurs southwest of the rat. The characterization of these cells as having a ‘boundary-off’ correlate, however, is best seen in the upright-barrier insertion manipulation already described above (figures 1 and 2). Following this manipulation (figure 6, middle row of firing rate maps), a boundary-off cell shows an additional region of low firing predicted by the BVC model, assuming that the cell represents the inverse of a BVC. Thus, for instance, cells 1 and 2 now exhibit a low-firing zone on the northeast side of the barrier, as if the cells were being inhibited by a BVC which fires when a boundary occurs proximally southwest of the rat. Even though the barrier is imperfectly oriented for cell 4 (north-to-south instead of northwest-to-southeast), the barrier clearly elicits an additional zone of low firing on the expected side of the barrier. The additional, barrier-elicited zones of low firing manifest in the square-walled environments (right columns of firing rate maps), as well as the circular-walled ones. In summary, our results suggest that these cells are best characterized as being like inverse short-range BVCs and we call these boundary-off cells. Although we have recorded only a few boundary-off cells to date, we note that they show relatively low theta modulation (figure 6, see 500 ms autocorrelogram, bottom row). While the spatial correlates of boundary-off cells appear fairly uniform, they may not consist of a single cell type anatomically and physiologically. For instance, although it is not trivial to infer cell types from waveforms, the waveforms of cells 1 and 4 are suggestive of interneurons, while the waveforms of cells 3 and 5 are suggestive of principal cells.

### (e) Quantification of boundary vector cells’ temporal and spatial properties and comparison to HD cells

Electronic supplementary material, figures S1 and S2 summarize key temporal and spatial properties of the entire BVC and HD cell sample in our experiment. BVCs (*n* = 46) exhibited significantly higher global mean rates (3.1 ± 0.3 Hz) than HD cells (*n* = 30; 1.3 ± 0.2 Hz; *t*_69.80_ = 4.78, *p* < 10^–5^) and were significantly more theta modulated (11.96 ± 1.84) than HD cells (*n* = 30; 4.96 ± 1.24; *t*_72.16_ = 3.16, *p* = 0.002).

As originally described in the BVC model, BVCs were expected to show no directionality in their firing fields. Thus, a given BVC might fire wherever there is a boundary 5 cm to the south of the rat, but irrespective of whether the rat is facing southwards (towards the boundary) or northwards (away from the boundary) and so on. Inspection of polar plots in the electronic supplementary material, figure S1 shows that most BVCs do indeed show very little directional modulation. Consistent with this expectation, HD cells showed significantly higher directional information rates (1.17 ± 0.18 bits s^−1^) than BVCs (0.14 ± 0.03 bits s^−1^, *t*_31.97_ = 8.77, *p* < 10^−9^), and directional selectivity was significantly higher for HD cells (6.51 ± 0.52) than for BVCs (1.88 ± 0.12; *t*_31.99_ = 8.77, *p* < 10^−9^).

In our previous quantification of subicular boundary cells’ spatial properties [17], we showed that BVCs carried more locational than directional information. We replicate that finding here with a new sample. Using procedures that aid removal of sources of bias from comparison of locational and directional signalling (see §2*g*), we found that BVCs exhibited a significantly higher locational than directional information rate. The estimated mutual information in bits per second was significantly higher between firing rate and location (0.40 ± 0.04) than between firing rate and direction (0.16 ± 0.03; *t*_45_ = 5.01, *p* = 0.000009). Consistent with this, BVCs show much higher locational selectivity (5.47 ± 0.96) than directional selectivity of firing (1.76 ± 0.11: *t*_45_ = 3.99, *p* = 0.0002).

In our previous work [17], the BVC sample consisted of presumptive principal cells only: we did not analyse other cells. In this report, we define all cells which exhibited the second field in the upright-barrier experiment (figure 2) as BVCs. Electronic supplementary material, figure S1 shows the waveforms for all the 46 BVCs in our sample. Appropriate classification should await a larger sample. For now, we note that some BVCs have waveforms with short peak-to-trough intervals and that some of those show high theta modulation (e.g. electronic supplementary material, figure S1, cells 26, 29, 39), suggestive that at least part of the BVC sample consists of interneurons.

## 4. Discussion

We have shown that a high proportion of cells in the subiculum, which have a firing field at/near the periphery of the environment, exhibit an additional firing field in a location predicted by the BVC model when an internal barrier is added to the environment in an appropriate orientation (i.e. perpendicular to the preferred allocentric direction of the cell as inferred from its firing without the barrier). Thus, for these cells at least, the perimeter of the testing environment (despite its familiarity) has no special status as a sensory determinant of spatial firing beyond other barriers to movement. The reliability of the field-repetition phenomenon strongly suggests that characterizing such cells as BVCs succinctly captures the spatial responses of these cells. Although this barrier-elicited field repetition was a strongly expected finding, our previous report of BVCs [17] emphasized the ‘non-remapped’ firing characteristics of subicular boundary cells across several different environments and showed only three examples of barrier-elicited additional fields. Accordingly, characterizing over 40 such examples in this report is a useful confirmation and extension of the BVC phenomenon. Furthermore, we show that field repetition of the kind predicted by the BVC model extends to drop-type boundaries. In our previous report [17], we had employed the ‘together–apart’ manipulation in one cell only (which did show the predicted field-repetition effect). Thus, the reliability of the field-repetition phenomenon in drop-type boundaries in all nine cells tested here provides further evidence of the validity of the BVC characterization of subicular boundary cells. Taking both sets of findings together, our report strongly suggests that these cells are specialized to code for environmental boundaries irrespective of their sensory nature. Finally, an additional contribution of this study has been to show that some cells with BVC properties including field repetition are likely to be interneurons.

The concept of an environmental boundary is somewhat abstract. Our best working definition of a boundary as inferred from rat BVC responses is that a boundary presents a behaviourally significant obstacle to locomotion along a broadly horizontal planar surface. It is important to note that such a boundary *need not actually prevent movement* to be effective as a boundary stimulus. For instance, our rats were fully able to cross the 10 cm gap by themselves in the ‘apart’ configuration of the ‘together–apart’ manipulation. Moreover, increasing exposure to the ‘apart’ configuration meant that the rats moved across the gaps with greater facility. Nevertheless, the BVCs still showed field repetition in the ‘apart’ configuration.

A related question concerns how boundaries are detected: what kind of sensory information defines the presence (and distance) of a boundary? In our simple initial models of how BVC firing is derived from environmental input to drive place cell firing [8], we assumed that the visual angle down from horizontal to the contrast provided by the wall meeting the floor provided an estimate for the distance to a boundary. Such a simple coding scheme would work equally well for a drop as for a wall (the angle to the end of the ground plane being the key measure). However, we have also shown BVC responses in the dark [17], ruling out a purely visual basis for BVC firing. In these examples in the dark, the BVCs responded at short range, so that tactile cues were available. It remains to be seen whether and how longer range BVCs fire in the dark. We are currently working to further understand what BVCs treat as a boundary, and how boundaries are detected, by probing the minimum conditions required to elicit field repetition.

Interestingly, we see no indication that BVC field repetition requires experience in the specific environments in which the field repetition is seen. As far as we can tell, a predictable BVC response to newly created environmental boundaries occurs immediately (e.g. figure 5*a*,*b*). However, it is an open question as to what extent the similarity of BVC responses to walled and drop-type boundaries, including field repetition to both, depends upon early developmental experience of different types of boundaries. Further, we have seen no sign in our BVCs that one or more fields weaken and/or disappear after environmental experience (e.g. figure 5*c*,*d*). This stands in contrast to observations of the plasticity of place cell responses to barrier insertion: after place field doubling in response to an inserted barrier, the ‘duplicate’ or original field often disappears with experience [7,14,16]. This contrast merits further confirmation, but invites the characterization of BVCs as spatial perceptual cells. It may be that in addition to using the BVC model to generate cell-specific predictions of firing patterns under environmental manipulation, a complete definition of cells as BVCs requires repeated testing.

### (a) Boundary-related inhibition

Boundary-off cells convincingly illustrate that the inhibition of firing at or near a barrier is a real physiological phenomenon. The mechanism seems simple enough to postulate. An inhibitory BVC will tend to shut down firing in a restricted zone near a barrier. Consistent with this, a boundary-off cell can be modelled as a cell that fires ubiquitously except in that restricted zone of inhibition provided by afferent inhibitory BVCs. As briefly set out in §1, excitatory boundary cells likely provide input to place cells and could stabilize grid cells indirectly via the place cells [16,17,32], and/or could help to stabilize grids directly [22,33]. However, what is the function of inhibitory BVCs and boundary-off cells?

One possibility is that inhibitory BVCs and excitatory boundary-off cells have different projections from excitatory BVCs and play a role in directing navigation. Many models of navigation require inhibited firing near barriers-to-movement in the interests of efficient locomotion, and this requirement is most obvious during ‘detour’ behaviour [34–36]. Subiculum has a strong projection to regions, for example the ventral striatum, which lie at the interface between spatial and motor systems. Accordingly, the joint action of inhibitory BVCs and excitatory boundary-off cells could be to promote motor sequences that avoid obstacles. Another potential function of inhibitory BVCs is suggested by a speculative model of grid cells outlined in this issue [37] in which grid cells are formed from inputs involving place and boundary interactions, and boundary cells contribute to a repulsive force which declines as a function of distance from environmental boundaries.

In summary, we show that a clear majority of subicular cells with fields near the perimeter of walled environments are well characterized as BVCs, that most BVCs treat walls and drop-type boundaries similarly (showing predictable, stable field repetition in response to both types of boundary) and that subicular cells exist (‘boundary-off’ cells), which are well described as the inverse of short-range BVCs. This study contributes to our understanding of how information from the external environment contributes to spatial mapping. How external-boundary-derived and self-motion-derived information combine and interact will be an important question for future study.
